# Characterizing headache patients admitted from the emergency department: a retrospective study

**DOI:** 10.3389/fneur.2024.1438312

**Published:** 2024-11-27

**Authors:** Faisal AlGhamdi, Abdulelah A. Alzahrani, Khaleel I. Alwatyan, Reem A. Hariri, Abdullah A. Alhowaish, Rahaf F. Almobarak, Mohammed Almulhim, Faisal B. Alkhadra

**Affiliations:** ^1^King Fahd University Hospital, Imam Abdulrahman Bin Faisal University, Al Khobar, Saudi Arabia; ^2^College of Medicine, Imam Abdulrahman Bin Faisal University, Dammam, Saudi Arabia

**Keywords:** headache, emergency department, demographics, clinical characteristics, outcomes

## Abstract

**Background:**

Headaches are a common complaint in emergency department (ED) presentations, but the demographics, clinical characteristics, and outcomes of patients admitted with headaches remain understudied. This retrospective study aims to investigate patients admitted with a chief complaint of headache.

**Methods:**

The study examined the triage database of our ED from 01/01/2020 to 31/12/2022 to identify patients admitted to either the wards or intensive care unit (ICU) with headache as their primary complaint.

**Results:**

Out of 347 identified patients, 100 met the inclusion criteria. The gender distribution was equal. The majority of cases (36%) were between 31 and 45 years old. Headache and dizziness were the most common complaints (54%), followed by chest pain (18%) and abdominal pain (10%). Neurological system involvement was observed in 60% of cases. Computed Tomography (CT) scans were performed in 87% of cases, while Magnetic Resonance Imaging (MRI) scans were done in 45% of cases. Comorbidities such as central nervous system (CNS) diseases (40%) and cardiovascular diseases (CVD) (36%) were prevalent. The average length of stay in the ED before admission was 35.3 h.

**Conclusion:**

This study provides insights into the demographics, clinical characteristics, and outcomes of patients admitted with headaches in the ED. The equal gender distribution and age distribution align with findings from other studies. The high utilization rate of CT scans suggests diagnostic uncertainty among emergency physicians. These findings contribute to the understanding of headache presentations in the ED and serve as a foundation for future research.

## Introduction

In 2021, World Health Organization (WHO) estimates that two in five people suffer from primary headache disorder worldwide most of them being female. This equates to 3.1 billion people globally (1). Despite such a high number, these headaches only account for roughly 1–2% of all ED visits worldwide (2). If we were to include secondary causes of headache the percentage would range from 1 to 4% of all ED visits (3–6). Primary headache disorder makes up the majority of headache complaints in the ED; however, secondary causes of headaches such as intracranial hemorrhage, infection, space occupying lesions like tumors or cysts must not be overlooked. Primary headache disorder, although debilitating, are relatively non-life threatening and most patients visiting the ED will be discharged home; secondary headache disorder presents a complex and life threating problem that if not acted upon rapidly could result in devastating consequences (2). In order to rule out these critical differential diagnoses, we call upon the use of neurological imaging specifically in the form of non-contrast head CT. This presents another problem in the form of neuroimaging overuse with increased dependence on imaging over clinical judgment (7).

Currently, the population of the Kingdom of Saudi Arabia sits just over 38 million (8). In 2022 the population of the eastern province was 5.1 million and has been steadily increasing since 2010 at a rate of 5% (±0.5%) per year (9). With the local population having a positive trajectory, this translates to increased visits to the ED. This retrospective study is designed to take a closer look at visits to our emergency department with the chief complaint of headache over a period of 2 years (from 1/1/2020 to 31/12/2022).

## Materials and methods

For this retrospective study, we conducted a thorough examination of the triage database at our institution from 1/1/2020 till 31/12/2022 to identify patients admitted from the emergency department to either the wards or the intensive care unit with headaches listed as their chief complaint. Inclusion criteria encompassed patients with headache documented as their primary complaint upon admission, admission to either the wards or ICU, and availability of a complete medical profile for analysis.

We gathered the following data, organizing it into three main categories: demographics, clinical characteristics, and headache-related characteristics. Under demographics, we documented the sex, nationality, and age of each patient. Clinical characteristics encompassed the triage given to each patient, the system involved (such as nervous system or cardiovascular system), and the final diagnosis upon admission. Within the headache-related characteristics category, we recorded details such as the onset and duration of headaches, severity of pain, associated signs and symptoms, comorbidities, diagnostic images obtained (such as MRI or CT scans), whether surgery was performed, consultation with neurology, outcomes of treatment (including headache resolution or improvement), and the length of stay in the hospital.

## Results

**Figure 1:**
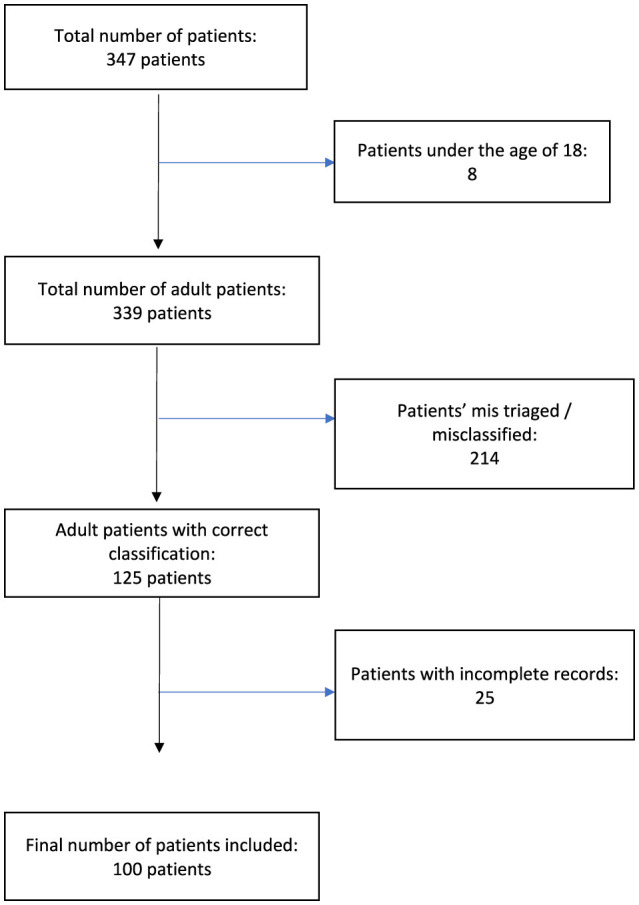
Flowchart of patients included in the study.

Out of the initial pool of 347 patients identified from the triage database, a total of 247 patients were excluded from the study based on predefined criteria. Specifically, 214 patients were excluded due to inaccurate triage classification regarding headache as their chief complaint. Twenty-five patients were excluded because their headaches were not adequately described in the medical records, while eight patients were under the age of 18 and therefore did not meet the inclusion criteria for adult patients. Hence, 100 patients were included. See Figure 1.

A total of 50 (50%) were females and 50 (50%) males. Majority 72% of cases were Saudi, average standard deviation (SD) age of patients was 43.5 (16) years. Majority 36% of cases had age between 31 and 45 years (Table 1).

**Table 1:** Demographics of patients (*n* = 100).

		**Frequency**
Sex	Male	50
	Female	50
Nationality	Saudi	72
	Non-Saudi	28
Age	≤ 30	24
Mean (SD) = 43.5 (16)	31–45	36
	46–60	27
	>60	13

Clinical characteristics are presented in Table 2. Neurological system was involved in 60% cases followed by Hematology/Infectious disease 9% cases, oncology in 8% cases.

**Table 2:** Clinical characteristics of patients (*n* = 100).

		**Frequency**
Triage	II	16
	III	28
	IV	47
	V	9
System involved	Neurology	60
	Cardiovascular	6
	Vascular	5
	Oncology	8
	Hematology/infectious disease	9
	Obstetrics and gynecology	3
	Endocrine	3
	Others	6
Final diagnosis	Cerebral venous sinus thrombosis	8
	Hypertension and diabetes	8
	Subarachnoid hemorrhage	9
	Ischemic stroke	11
	Interventricular hemorrhage	5
	Headache	6
	Iron deficiency anemia	7
	Left temporal glioblastoma	5
	Epidural hematoma	4
	Ventriculoperitoneal malfunction	3
	Intracranial tumor	4
	Others [^*^]	30

^*^Astrocytoma, biliary acute pancreatitis, brain lesion, breakthrough seizure, cervical discopathy, chronic kidney disease post-dialysis, clinoidial meningioma, colloid cyst, COVID-19 Pneumonia, demyelinating disease, developmental arteriovenous fistula, dural arterial venous malformation, encephalitis, frontal lobe lesion, functional neurological disorder, hirayama disease, internal carotid artery aneurysm, meningoencephalitis, pituitary macroadenoma, post craiectomy wound infection, pseudotumor cerebri, right medial sphenoid wing meningioma, sepsis secondary to mitral valve infective endocarditis, suprasellar lesion, seizure, urinary tract infection, vertigo, and viral encephalitis.

Headache related characteristics, management, and outcomes are presented in Table 3. In majority 32% of cases onset of headache was more than 7 days followed by < 1 day in 21% cases, 1–3 days in 11% cases. Majority 48% of cases had headache since < 7 days followed by 7–30 days in 20% cases. Out total, 22% had moderate pain, 16% had mild and 16% had severe headache while 46% did not reported the severity. Nausea was the most common symptoms associated with headaches found in 44% cases followed by dizziness in 32% cases, Photophobia in 12% cases, Diplopia in 9% cases, trauma in 7% and fever in 5% cases. Cardiovascular diseases (CVD) were the most common comorbidities found along with headache patients found in 36% cases followed by CNS in 34% cases, Endocrine disorders in 25% cases. CT scan was done in 87% cases while MRI was done in 45% cases. Sixty six percent cases involved consultation with neurology while surgery was done in 24% cases. Out of 100, 60% cases were stable; the condition of 38% cases was improved while 2% were died. Average (SD) length of stay was 35.3 (6.6) h in the emergency department before admission.

**Table 3:** Headache related characteristics, management, and outcomes of patients (*n* = 100).

		**Frequency**
Headache onset	< 1 day	21
	1–3 days	11
	3–7 days	4
	>7 days	32
	Not mentioned	32
Headache duration	< 7 days	48
	7–30 days	20
	>30 days	13
	Not mentioned	19
Severity of pain	Mild	16
	Moderate	22
	Severe	16
	Not mentioned	46
Sign and symptoms	Fever	5
	Nausea	44
	Dizziness	32
	Photophobia	12
	Diplopia	9
	Trauma	7
Comorbidities	CVD	36
	CNS	34
	Migraine	6
	Endocrine disorders	25
	Psychological disorders	2
Diagnostic images	CT	87
	MRI	45
Management	Neurology consulted	66
	Surgery done	24
Outcomes	Stable	60
	Improved	38
	Died	2

## Discussion

In the context of emergency department presentations, headaches represent a common yet multifaceted clinical challenge, often requiring prompt assessment and management, sometimes even admission (2–6). In this study, we sought to outline the demographics, clinical characteristics, and therapeutic outcomes of patients admitted from the ED with headaches as their chief complaint.

The examination of the relationship between patient demographics such as age and gender yield several significant insights. Available literature has shown that the overall prevalence of headaches is markedly higher among females, with one study reporting a significant majority of headache presentations in emergency departments being females (77.8%) and another finding 76% of patients presenting with headaches being women, with a mean age of 37 years (10, 11). This trend is consistent across various demographics, suggesting a gender-specific predisposition to headache disorders.

Notably, our study revealed an equal gender distribution among these patients, which diverges from previous findings reported in various regions including Saudi Arabia (7), Italy and Austria (12), and Germany (13), but is congruent with the incidence of severe headache in a study conducted in the United States (14). One plausible explanation for this disparity could stem from the relatively small sample size of the cohort. Another reason could be due to the broad inclusion criteria and lack of focus on headache subtypes that are more gender prevalent such as migraines in women and cluster headaches in men. The majority (36%) of cases fell within the age range of 31–45 years, with an average age of 43.5 years. This age distribution aligns with findings from other studies, such as those conducted in Singapore (15) and Europe (12), where mean ages of 41 and 43.32 years, respectively, were reported.

In the context of emergency department neuroimaging, the non-enhanced head CT scan emerges as a favored modality (16). During our investigation period, we observed a notably elevated utilization rate of brain CT scans, reaching 87% among patients. This figure contrasts sharply with proportions reported in prior similar studies, where CT scans were employed in 21.8, 28.8, and 39.2% of cases (13, 17, 18). The disparity in utilization rates could be attributed to several factors. Firstly, there exists a notable diagnostic uncertainty among emergency physicians when managing patients presenting with headaches, which was the most common reason for admission as demonstrated by a study conducted at the National University Hospital of Singapore (15). Secondly, the accessibility of round-the-clock emergency diagnostic services offering CT and MRI scans may contribute to the heightened utilization observed. Despite MRI scans being utilized to a lesser extent compared to CT scans, with a frequency of 45% in our study, this still represents a substantial proportion. This prevalence is notable considering that many guidelines for headache diagnosis recommend imaging only in cases where the patient's history or neurological examination indicates the possibility of an underlying secondary pathology (19). A possible explanation behind the low threshold for imaging in our cohort could be the fear of medicolegal consequences that may arises with misdiagnosis of life-threatening conditions.

For further understanding of the possible relationship between the clinical assessment of headaches with past medical history, the prevalence of certain comorbidities in patients presenting with headache was calculated. In our analysis, central nervous system diseases were particularly the most common established conditions (40%) which corresponds with a prospective observational study in Australia and a cross sectional study in Germany (13, 20). This is particularly important as 11% of patients in our cohort who initially presented with headaches as their complaint were found to be having ischemic strokes. This is similar to the Rimmele et al. study which found that strokes compromised 20.8% of secondary headaches (13). This raises concerns as strokes may manifest as tension-like pattern headaches resulting in their under diagnosis as they may be deemed less concerning (21).

Furthermore, cardiovascular diseases were the second most frequent comorbidities (36%) followed by endocrine conditions (25%), which is precisely parallel with the study in Germany as 22.9 and 12.8% presented with known cardiovascular system (CVS) disease and endocrine conditions, respectively (13). In this current study, only 2% had a pre-existing history of psychological disorders which diverges from recent literature where a study in Iran showed a considerable percentage of subjects (80%) had depression (22). The disparity can be attributed to missed diagnoses in our sample and underutilization of depression screening tools. Moreover, this indicates the importance of comprehensive and multi-disciplinary approach to headache as it encompasses multiple systems.

Diagnosis of headaches within the emergency setting remains a difficult task regardless of experience or availability of services and equipment. Even though we excluded undiagnosed and under investigated patients, finding a specific diagnosis remains a common issue with these patient groups in the ED. An Italian study following up on patients discharged from the ED with the label of not otherwise specific (NOS) headaches found that emergency physicians were capable of diagnosis only 37% of these headaches but failed to mention so in their discharge reports (23). Another study also found similar results when comparing between patients discharged from the ED and following up in their institution's headache unit. They found that the concordance between the two was low and proposed that this could be due to the inherent factors associated with patients presenting to the ED such as life-threatening cases, shorter time to take history and overcrowding of the ED (24).

Some issues warrant comments. First, the reliance on a triage database for patient selection introduces the possibility of missed cases, potentially leading to underrepresentation and selection bias. Given the retrospective nature of the study, some data may not have been consistently documented, particularly regarding specific headache characteristics such as onset, duration, and severity. Furthermore, the retrospective design limits the ability to establish causality or temporality between variables. Additionally, our cohort was recruited during the COVID-19 pandemic this could have led to under representation of the population as patients with headache may avoid hospitals due to fear of infection. Lastly, the triage process in our institution is done by nurses and could lead to mis triaging of serious cases and delaying patient care. Despite these limitations, this study provides insights into the demographics, clinical characteristics, and outcomes of patients admitted with headaches from the ED serving as a foundation for future research in this area.

In conclusion, our study of 100 patients admitted from the emergency department with headaches as their chief complaint reveals important insights into demographic distribution and clinical characteristics. The study population consisted of an equal distribution of males and females, with a majority being Saudi nationals and a mean age of 43.5 years. Headache and dizziness were the most common complaints, involving the neurological system in the majority of cases. These findings showcase the diverse nature of headache presentations in the emergency department and provide a foothold for future research.

## Data Availability

The raw data supporting the conclusions of this article will be made available by the authors, without undue reservation.
